# Differences in the efficiency of 3-deazathiamine and oxythiamine pyrophosphates as inhibitors of pyruvate dehydrogenase complex and growth of HeLa cells *in vitro*

**DOI:** 10.1080/14756366.2020.1844681

**Published:** 2020-11-13

**Authors:** Ewa Grabowska, Magdalena Czerniecka, Urszula Czyżewska, Aneta Zambrzycka, Zenon Łotowski, Adam Tylicki

**Affiliations:** a Doctoral School of Exact and Natural Sciences, University of Białystok, Białystok, Poland; b Department of Microbiology and Biotechnology, University of Białystok, Białystok, Poland; c Argenta Sp. z o.o, Poznań, Poland; d Department of Organic Chemistry, University of Białystok, Białystok, Poland

**Keywords:** Apoptosis, thiamine antimetabolites, thiamine pyrophosphate-dependent enzyme inhibitors

## Abstract

Oxythiamine (OT) and 3-deazathiamine (DAT) are the antimetabolites of thiamine. The aim of study was to compare the effects of OT and DAT pyrophosphates (-PP) on the kinetics of mammalian pyruvate dehydrogenase complex (PDHC) and the *in vitro* culture of HeLa cells. The kinetic study showed that 3-deazathiamine pyrophosphate (DATPP) was a much stronger competitive inhibitor (*K_i_* = 0.0026 μM) of PDHC than OTPP (*K_i_* = 0.025 μM). Both *K_i_* values were much lower versus *K*_m_ for thiamine pyrophosphate (0.06 μM). However, DATPP added to the culture medium for the HeLa cells culture did not hamper the rate of cell growth and showed not significant impact on the viability of the cells, whereas OTPP and OT showed a significant cytostatic effect. The differences between the thiamine antivitamins in their effect on cell growth *in vitro* may be due to differences in physicochemical properties and difficulty in DAT transport across the cell membrane.

## Introduction

**Figure 1.**
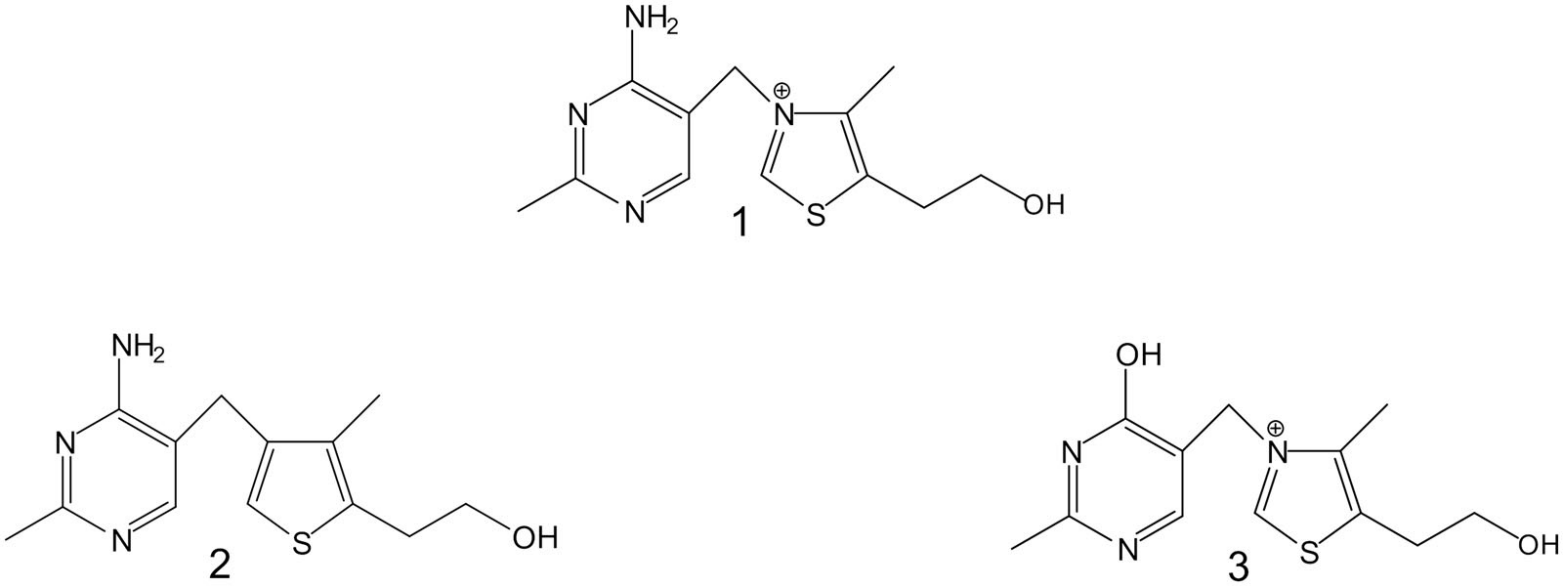
Structure of thiamine (**1**), 3-deazathiamine (**2**) and oxythiamine (**3**).

Thiamine (Figure 1, No. **1**) is one of the most important vitamins needed for proper cell metabolism. It performs several functions, of which the main is its role as a cofactor of important enzymes, such as pyruvate dehydrogenase complex (PDHC), transketolase, 2-oxoglutarate dehydrogenase complex and pyruvate decarboxylase^1^^,^^2^. For many years, several antimetabolites of thiamine [such as amprolium, metronidazole, pyrithiamine or oxythiamine (OT)] have been synthesised and tested as antibiotics or cytostatics^3–6^.

3-Deazathiamine (DAT, Figure 1, No. **2**) is a compound known from the beginning of the 21st century. It was synthesised for the first time by Hawksley et al.^7^. The main difference between thiamine and this antivitamin is that it lacks the N3 cation in the thiazolium ring, which is replaced by a carbon atom. This modification prevents the formation of ylide and thus affects the catalysis after the incorporation of the derivative into the active centre of thiamine pyrophosphate (TPP) dependent enzymes. Currently, this compound is synthesised in two ways. The first one involves the use of 2-acetylbutyrolactone as a substrate for the formation of thiazolium ring. Pyrimidine ring is synthesised from acetamidine hydrochlorine and 3-anilinopropionitrile^8^. In the second way, the same substrate is used for pyrimidine ring synthesis, but 3-methylthiophene is used as the first compound for the formation of thiazolium ring^9^^,^^10^. Studies on the impact of the DAT pyrophosphate (DATPP) on the activity of TPP-dependent enzymes have been done on *Zymomonas mobilis* pyruvate decarboxylase (25000 times stronger binding than TPP) and *Escherichia coli* 2-oxoglutarate dehydrogenase complex (about 500 times stronger binding than TPP) only^11^. Despite the great importance of PDHC in cell metabolism, no data are available showing the effect of DAT on the parameters of the enzymes from mammalian cells. Similarly, despite the proven inhibitory properties of DAT on the above mentioned TPP-dependent enzymes from bacteria^11^ (properties that may indicate a potential cytostatic effect of this derivative), there is no information in the literature about the interaction of DAT with cell *in vitro* models.

OT (Figure 1, No. **3**), in contrast to DAT, is one of the best-known antivitamins of thiamine. Research done in 1984 on transketolase and PDHC isolated from rat adrenals showed that the enzymes are inhibited in the presence of OT^12^. Most importantly, another study^13^ showed four times lower activity of PDHC after the injection of OT (1 mM/kg of rats’ body weight), suggesting that this compound may be an inhibitor of TPP-dependent enzymes *in vivo*. Moreover, OT has been assumed to inhibit the growth of cancer cells. A study done on PC-12 cells showed over 90% inhibition of cell growth (depending on the concentration and duration of incubation)^14^. Research performed on two models, *Caco-2*^15^ cells and rat membrane vesicles^16–18^ showed that in the presence of OT, thiamine transport is reduced (in the case of rat membrane vesicles, from about 20% up to about 70%^16–18^). However, OT may have no effect on thiamine transport as well (as shown by a study on BeWo human trophoblasts^19^). In addition, OT also may decrease the cell viability of colon carcinoma^20^. The impact of OT was also studied on the cells of fungi, such as *Malassezia pachydermatis*, *Saccharomyces cerevisiae* and *Candida albicans*^5^^,^^6^^,^^21^. Research shows that OT can influence the lipid content in fungal cells^21^. It decreased the amount of polyunsaturated fatty acids in *C. albicans* cells, unlike *S. cerevisiae*^21^, while opposite results were shown for monounsaturated fatty acids^21^. Moreover, another research using *S. cerevisiae* cells which focussed on the activity of TPP dependent enzymes in the presence of OT showed that this compound may be a stronger inhibitor of mitochondrial enzymes (such as PDHC decrease by 50%) than the TPP-dependent enzymes present in the cytosol (such as pyruvate decarboxylase)^6^. A study done on *M. pachydermatis* showed that, among the several antivitamins of thiamine, OT has the strongest fungicidal effect^5^.

Based on the limited data available on the inhibitory properties of DAT in relation to some TPP-dependent enzymes isolated from microorganisms, it can be assumed that this antimetabolite should have similar properties in other organisms and other TPP-dependent enzymes like PDHC from mammals. The above assumption allows us to hypothesising that DAT, by inhibiting TPP-dependent enzymes, will reduce the rate of cell growth *in vitro* and limit cell viability. In this work, we decided to test the above hypothesis by comparing the effects of the well-known thiamine antivitamin OT with the properties of DAT against the PDHC isolated from the porcine heart. In addition, we compared the effects of the above-mentioned thiamine antivitamins on HeLa cancer cells *in vitro*. The obtained results will provide the basis for assessing the cytostatic properties of DAT against cancer cells.

## Materials and methods

### Sources of DAT, OT and their phosphate derivatives

Three compounds were analysed in the study – OT, OT pyrophosphate (OTPP) and 3-deazathiamine pyrophosphate (DATPP). OT was bought from Sigma Aldrich (catalog number: O400) and OTPP as well as DATPP was synthesised in the Faculty of Chemistry, University of Białystok, as described below.

**Figure 2.**
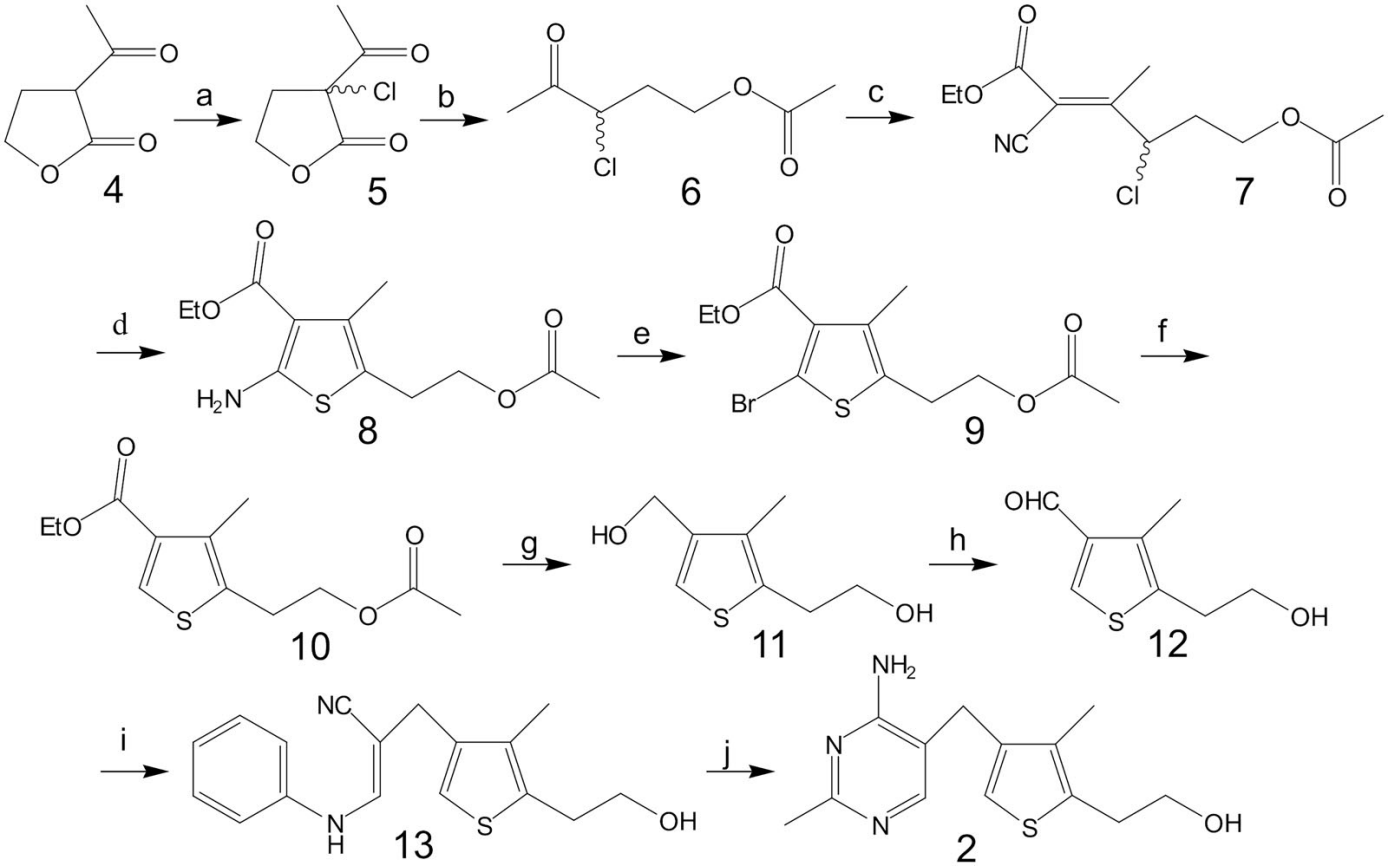
Preparation of 3-deazathiamine (according to Hawksley et al.^8^): (a) SO_2_Cl_2_; (b) 1) AcOH, HCl/H_2_O, 2) Ac_2_O; (c) NCCH_2_COOEt, AcONH_4_, PhMe; (d) NaSH, EtOH; (e) CuBr_2_, *t*-BuONO, CH_3_CN; (f) Zn, AcOH; (g) LiAlH_4_, Et_2_O; (h) MnO_2_, CHCl_3_; (i) PhNH(CH_2_)_2_CN, NaOMe, DMSO, MeOH; (j) CH_3_C(=NH)NH_2_·HCl, NaOEt, EtOH.

**Figure 3.**
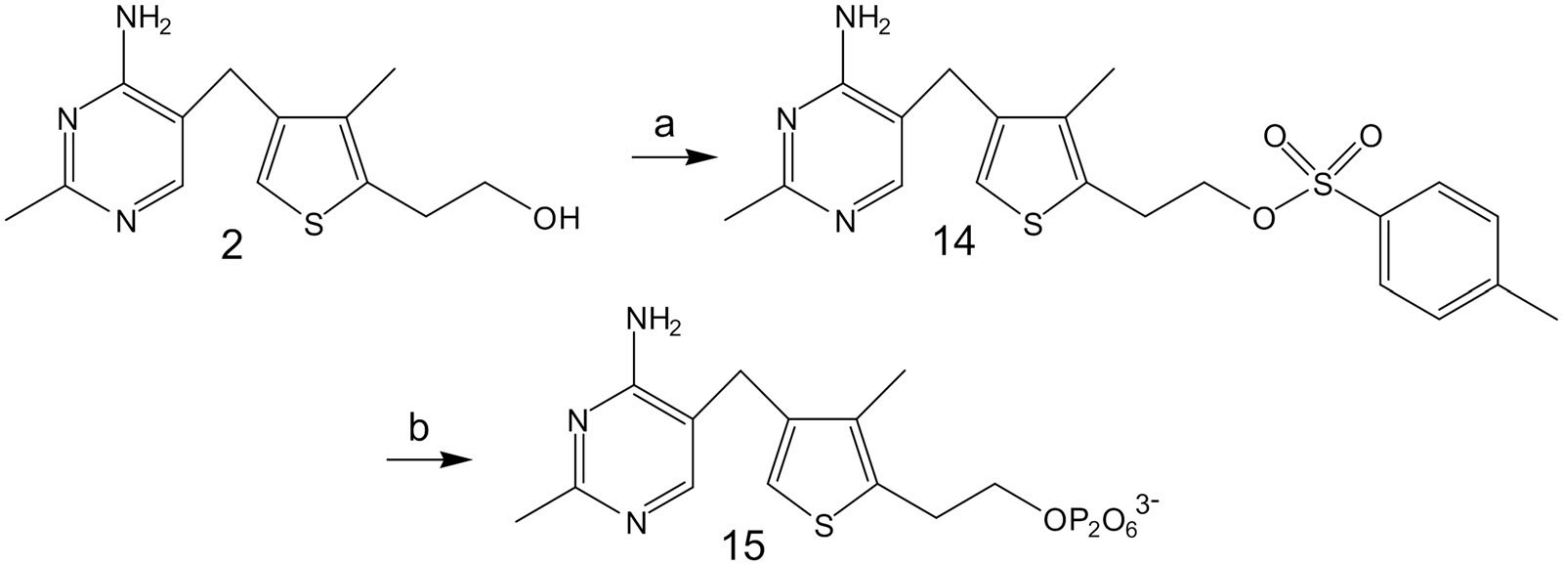
Preparation of 3-deazathiamine pyrophosphate (according to Zaho et al.^9^): (a) *p*-TsCl, py; (b) (Bu_4_N)_3_HP_2_O_7_, CH_3_CN.

DAT synthesis (Figure 1, No. **2**) was carried out according to the procedure described by Hawksley et al.^7^ (Figure 2). The transformation of DAT (Figure 3, No. **2**) into its pyrophosphate ester (Figure 3, No. **15**) was performed using the method described by Zhao et al.^9^. The crude product was purified by means of column chromatography on silica gel using methanol/water solvent (8:2 ratio, with 10 drops of ammonia water added for 100 ml of eluent). The collected fractions were lyophilised, and DATPP (Figure 3, **No. 15**) was obtained as a white powder.

**Figure 4.**
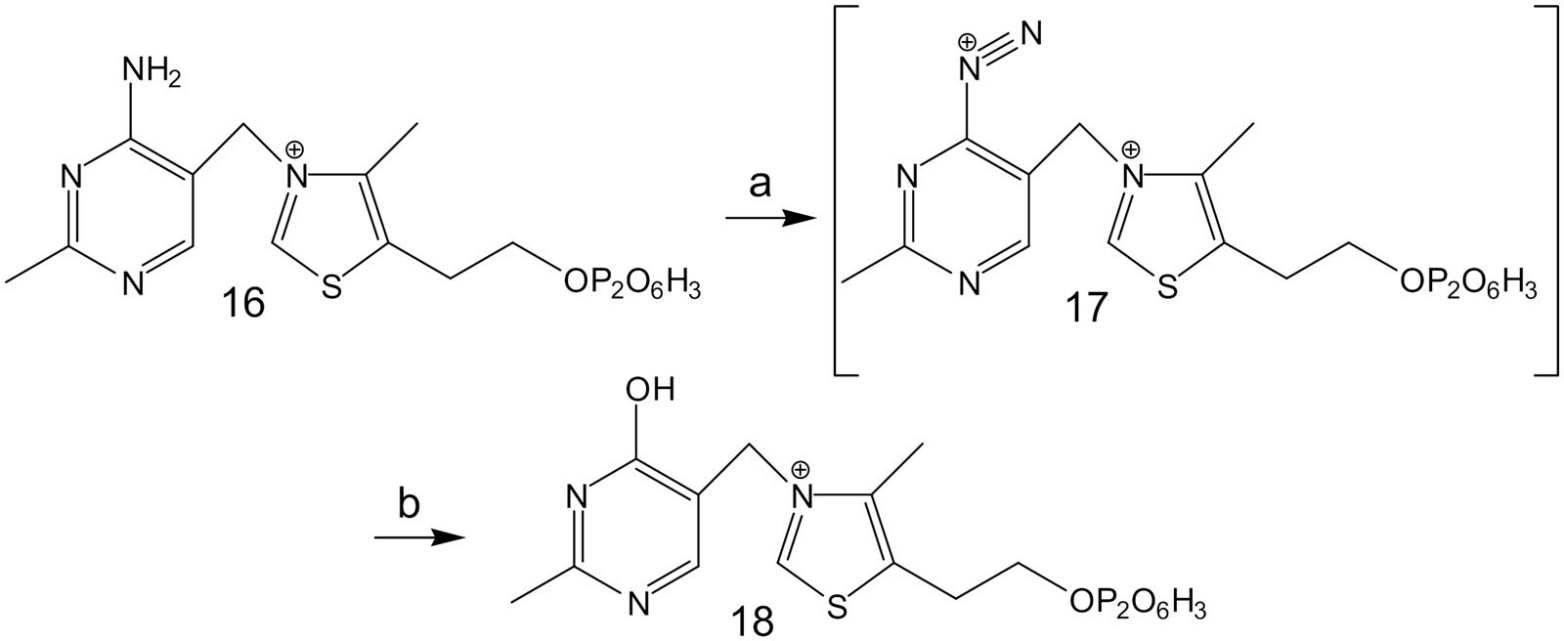
Preparation of oxythiamine pyrophosphate: (a) NO (HCl + NaNO_2_), air; (b) H_2_O.

OTPP (Figure 4, No. **18**) was obtained from TPP (Figure 4, No. **16**) in a Sandmeyer-type reaction, which involves the transformation of the amino group from the pyrimidine ring into diazonium salt (Figure 4, No. **17**; not isolated during the reaction) and conversion of this reactive entity to phenol with water^22^.

The NMR data of OTPP (Figure 4, No. **18**) were recorded on a Bruker Avance II spectrometer in D_2_O:

^1^H NMR (400 MHz, ref. DSS), δ: 9.69 (s, 1H, Ar-H from thiazolium ring), 7.96 (s, 1H, Ar-H from pyrimidine ring), 5.58 (s, 2H, Ar_1_-CH_2_-Ar_2_), 4.12 (m, 2H, Ar-CH_2_-CH_2_-O), 3.31 (m, 2H, Ar-CH_2_-CH_2_-O), 2.64 (s, 3H, CH_3_), 2.56 (s, 3H, CH_3_).

^13^C NMR (100 MHz, ref. DSS), δ: 168.6 (C), 165.6 (C), 154.3 (C + CH), 145.4 (C), 137.3 (CH), 118.0 (C), 66.1 (CH_2_), 52.9 (CH_2_), 30.5 (CH_2_), 23.3 (CH_3_), 13.8 (CH_3_).

^31^P NMR (162 MHz, ref. H_3_PO_4_), δ: −9.72 (dd, *J_1_*=7.8 Hz, *J_2_*=20.8 Hz, 1 P), −11.55 (d, *J* = 20.9 Hz, 1 P).

### Isolation of mitochondrial PDHC

The first part of the experiment was focussed on comparing the effects of OTPP and DATPP on the kinetic properties of PDHC. For this purpose, the enzyme was isolated from porcine heart according to the procedure desribed by Stanley and Perham ^23^ and modified by Strumilo et al.^24^. Briefly, porcine tissue was homogenised in a Teflon homogeniser for 3 min with 0.15 M KCl on ice (tissue to solution ratio 1:5). To isolate mitochondria, the cell homogenate was centrifuged for 7 min. at 600 × g, and then the obtained supernatant was centrifuged again for 10 min at 10,000 × g. To purify mitochondria, the pellet was resuspended three times with 0.3 M sucrose and centrifuged for 10 min at 10,000 × g. Finally, mitochondria were suspended with 0.025 M phosphate buffer (pH 7.5), in a 1:1 ratio. For the disintegration of mitochondria, the suspension was frozen in liquid N_2_ and thawed three times and then centrifuged for 40 min. 40,000× g. For PDHC precipitation supernatant was mixed with polyethylene glycol up to 36% and centrifuged 40 min at 40,000 × g. The precipitate was resuspended with 0.025 M phosphate buffer (pH 7.5) and used for enzymological studies.

### Measurements of the kinetic properties of PDHC

To determine the range of saturation of the obtained PDHC preparation with endogenous TPP, the enzyme activity was evaluated (procedure given below) with the addition of exogenous TPP (0.2 mM) without antivitamins in relation to evaluation without the addition of TPP in the reaction mixture. The results showed that the saturation of the preparation by endogenous TPP did not exceed 10%. Kinetic calculations were corrected to pure apoform.

For the estimation of *K*_m_ and *V*_max_ of TPP, a solution containing 2 mM pyruvate, 2 mM NAD^+^, 0.1 mM CoA, 1 mM Mg^2+^ and 1 mM DTT in 50 mM phosphate buffer (pH 7.8), and 10 µl of the PDHC preparation containing TPP at a concentration of 0.02 − 5.0 µM were used^24^^,^^25^. The changes in the reaction speed of PDHC were measured in the presence of inhibitors (OTPP or DATPP at a concentration of 0.01 µM) in the same reaction mixture. Measurements were done on Beckman DU-640 spectrophotometer (wavelength: 340 nm). Each measurement was repeated five times. The *K*_m_ values of coenzyme in the presence of both antivitamins were used for estimating the *K_i_* values of the inhibitors in relation to PDHC. For estimating the *K_i_* values, we used the formula given below.

$$K_{i}=\frac{\left[I\right]}{\frac{K_{m}^{i}}{K_{m}}-1}$$

where *K_i_* - inhibition constant; [*I*] - inhibitor’s concentration; $K_{\text{m}}^{i}$ - *K*_m_ of TPP in the presence of inhibitor; and *K*_m_ - Michaelis constant of TPP.

### Impact of antivitamins on *in vitro* cell culture

To evaluate the impact of the tested antivitamins on an *in vitro* cell model, HeLa cells were incubated in a CO_2_ incubator (37 °C, 5% CO_2_, 95% humidity). Three independent experiments were performed for statistical calculations. All the cultures were maintained in MEM199 medium, with 10% foetal bovine serum and antibiotics (penicillin 50 U/ml, streptomycin 50 µg/ml). Control cultures (without antivitamins) and experimental variants (with thiamine analogues OT, OTPP and DATPP, at a concentration of 0.005 − 0.02%) were grown until the control variant reached confluence (approximately 3–4 days).

The impact of the chosen thiamine analogues was evaluated by analysing the metabolic activity of cells by the MTT test, using Lambda E MWG AG BIOTECH plate reader. For assessing the number of live/dead cells as well as live/dead cells with division into early and late apoptosis Muse™ Count and Viability and Muse™ Annexin V & Dead Cell kits were used, according to the manufacturer’s instructions. To define those parameters of cells, Merck Millipore Muse™ Cell Analyser (0500–3115) was used.

### Statistical analysis

The results were statistically analysed using the Shapiro–Wilk *W*-test to identify normal distribution and Levene *L*-test for verifying if the variances were homoscedastic. In the case of the normal distribution of data and homoscedastic variances, the *t*-Student test was used to compare the mean values, while in the case of nonnormal distribution of data, nonparametric test (*U*-Mann–Whitney test) was used.

## Results

**Figure 5.**
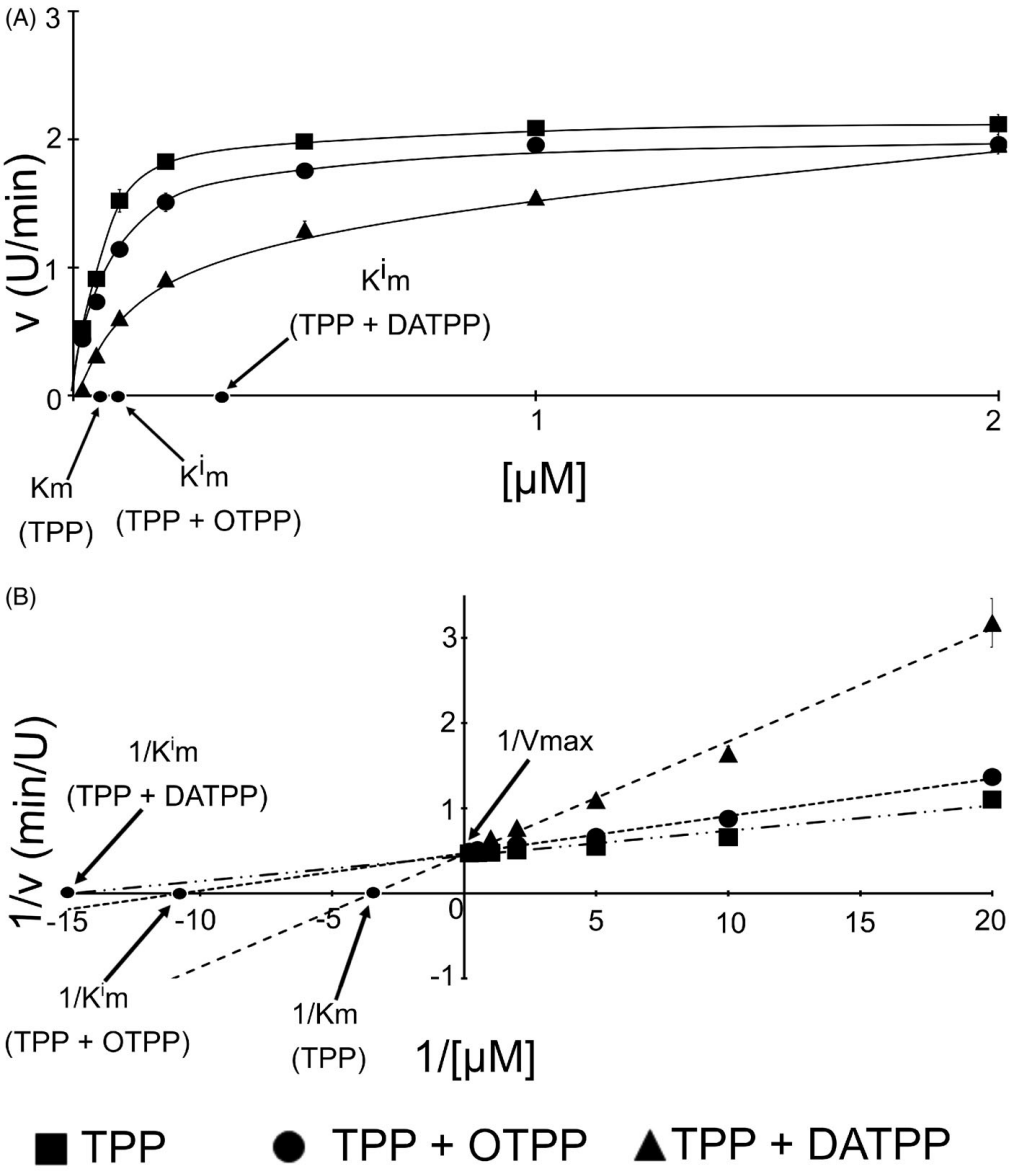
(A) Michaelis-Menten and (B) Lineweaver-Burk plots of pyruvate dehydrogenase complex properties in the presence of tested compounds (concentration of TPP: 0.02–5 µM; concentration of OTPP and DATPP: 0.01 µM). DATPP: 3-deazathiamine pyrophosphate; OTPP: oxythiamine pyrophosphate; TPP: thiamine pyrophosphate.

Measurements of the activity of PDHC in the presence of various concentrations of TPP and with or without tested antivitamins allow to preparation Michaelis Menten and Lineweaver Burk plots (Figure 5A,B). These plots showed that, in the presence of the natural coenzyme as well as anticoenzymes, PDHC exhibited hyperbolic kinetics. The addition of both tested anticoenzymes did not affect the *V*_max_ value but increased the $K_{\text{m}}^{i}$ of PDHC in comparison with the *K*_m_ of TPP. The Lineweaver Burk model was used to calculate the *K*_m_ and *V*_max_ values. The obtained data showed that OTPP and DATPP are competitive inhibitors of PDHC. Knowing the type of inhibition, we could determine the inhibition constant (*K_i_*) values for individual anticoenzymes. As it was shown in Table 1, the *K_m_* value of TPP was about two times higher than the *K_i_* value of OTPP and about 20 times higher than the *K_i_* value of DATPP (Table 1). Moreover, the *K_i_* value of OTPP was about 10 times higher than that of DATPP.

**Table 1.** Comparison of the *K*m of thiamine pyrophosphate (TPP), *K_i_* value of oxythiamine pyrophosphate (OTPP) or 3-deazathiamine pyrophosphate (DATPP), *K*_m_/*K_i_* ratio and *V*_max_ of pyruvate dehydrogenase complex

Coenzyme/ anticoenzyme	Kinetic parameters			
	*K*_m_ (µM)	*K_i_* (µM)	*K*_m_/*K_i_*	*V*_max_ (U/min)
TPP	0.06 ± 0.015	—	—	2.22 ± 0.088
OTPP	—	0.025 ± 0.015^a^	2.4	2.1 ± 0.063
DATPP	—	0.0026 ± 0.0009^a^^,b^	23.1	2.18 ± 0.42

^a^Statistically significant differences between the *K_m_* of TPP and the *K_i_* of OTPP or DATPP (*U*-Mann–Whitney test, *p* < 0.05).

^b^Statistically significant differences between the *K_i_* of OTT and DTPP (*U*-Mann–Whitney test, *p* < 0.05).

Experiments on *in vitro* cell culture were conducted to determine the amount of cells (dead/live), their metabolic activity (MTT test) and the intensity of apoptosis.

**Figure 6.**
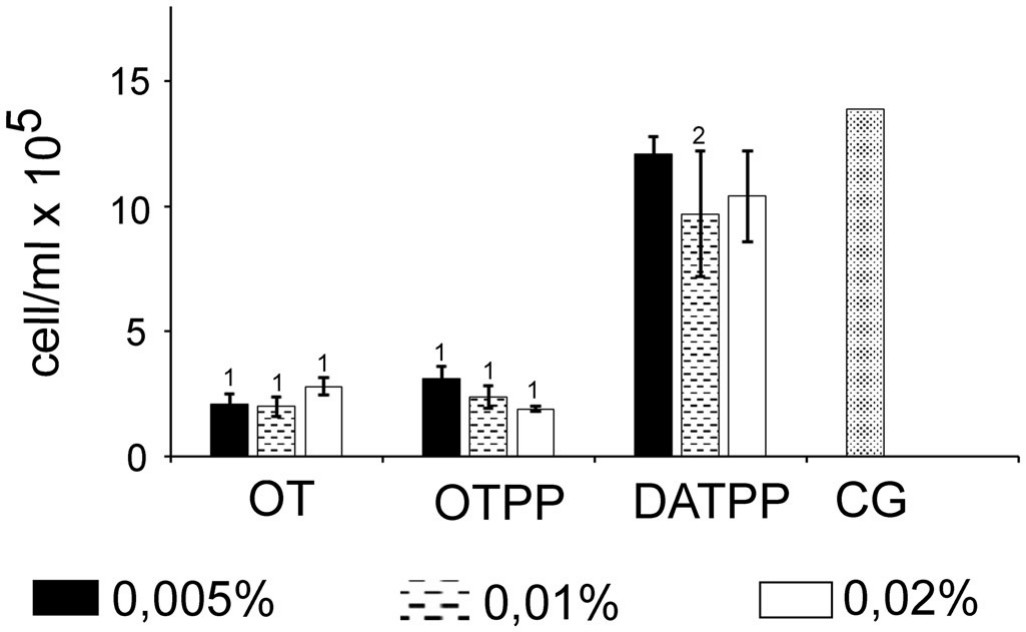
Comparison of the total amount of cells after 4 days of HeLa cells culture with different concentrations of antivitamins (0.005%, 0.01%, 0.02%). CG: control group; DATPP: 3-deazathiamine pyrophosphate; OT: oxythiamine; OTPP: oxythiamine pyrophosphate; statistically significant differences in relation to control group: ^1 ^*U*-Mann–Whitney test; ^2^*t*-Student test; *p* < 0.05.

A comparison of the number of cells in the presence of OT, OTPP or DATPP showed that the least effective thiamine analogue among the three derivatives tested was DATPP. OT and OTPP reduced the number of HeLa cells after 4 days of incubation by 80% compared with control (Figure 6). In the presence of DATPP, only a slight decrease in cell number was observed (Figure 6).

The MTT test was performed for all experimental variants which contained different concentrations of the tested thiamine antivitamins. The results showed that the most effective antivitamins were OT and OTPP. DATPP caused a decrease in the metabolic activity of HeLa cells as well, but to a lower extent (Table 2). After the incubation of HeLa cells with OT and OTPP, over 50% (concentration 0.005%) and over 60% (concentration 0.02%) decrease in metabolic activity were observed in comparison to control. On the other hand, after incubation with DATPP, the reduction in the metabolic activity of cells did not exceed 50%.

**Table 2.** Comparison of the results of MTT test for HeLa cells in the presence of oxythiamine (OT), oxythiamine pyrophosphate (OTPP), 3-deazathiamine pyrophosphate (DATPP), data are presented as percentage in relation to the control group including the number of cells in specific groups (as a percentage of control group)

	Concentration of antivitamin		
Antivitamin	0.005%	0.01%	0.02%
OT [% of the control group]	46^a^ ± 15	40^a^ ± 16	38^a^ ± 16
OTPP [% of the control group]	45^a^ ± 15	37^a^ ± 16	38^a^ ± 16
DATPP [% of control group]	54^a^ ± 12	59^a^ ± 12	51^a^ ± 11

^a^Statistically significant difference in comparison to the control group (*U*-Mann–Whitney test, *p* < 0.05).

**Figure 7.**
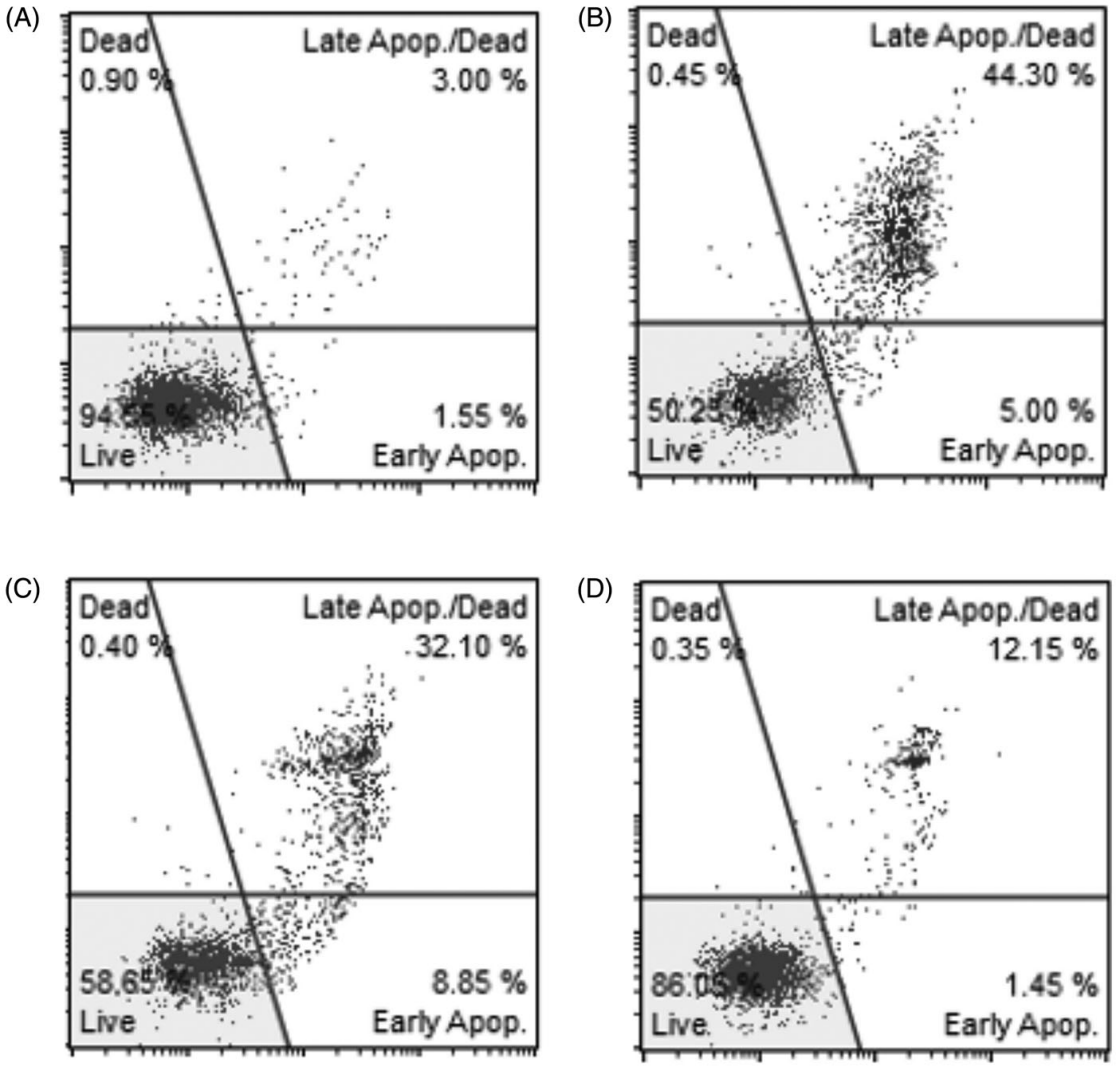
Viability of HeLa cells after 4 days of culture on (A) control medium, (B) oxythiamine-containing medium, (C) oxythiamine pyrophosphate-containing medium and (D) 3-deazathiamine pyrophosphate-containing medium. The concentration of all antivitamins was 0.02%.

**Figure 8.**
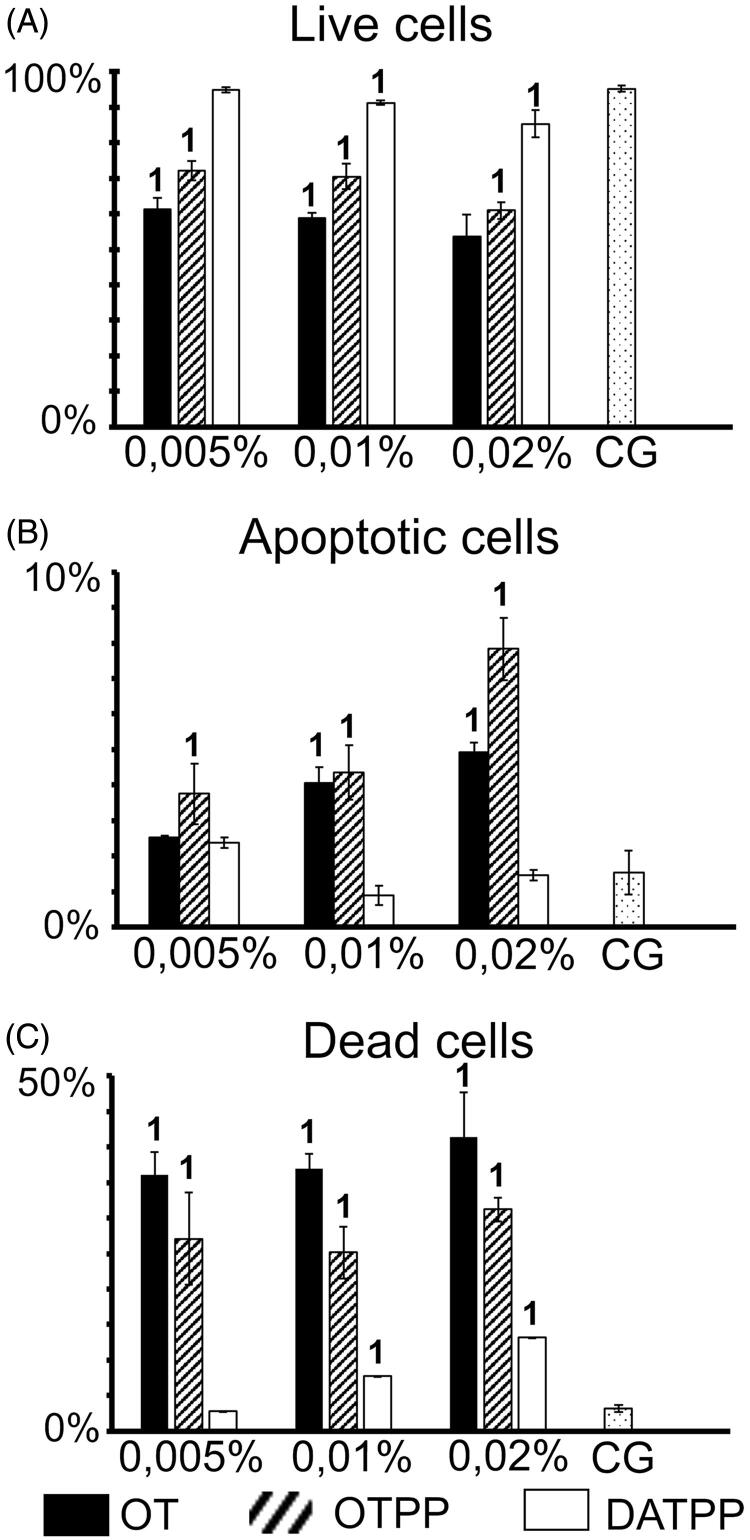
Comparison of the percentages of (A) live, (B) apoptotic and (C) dead HeLa cells after 4 days of incubation with thiamine antivitamins: oxythiamine (OT), oxythiamine pyrophosphate (OTPP), and 3-deazathiamine pyrophosphate (DATPP). Data compared with the control group (CG): ^1^statistically significant difference in comparison with the control group, *t*-Student test, *p* < 0.05; ^2^statistically significant difference in comparison with the control group, *U*-Mann–Whitney test, *p* < 0.05.

Sample results obtained by analysing the viability profile of HeLa control culture and after incubation with 0.02% of tested antivitamins are shown in Figure 7. Data obtained from three independent cultures for each antivitamin are shown in Figure 8, in which the changes in the percentage of live, dead, and apoptotic cells after incubation with different concentrations of antivitamins are clearly presented. Incubation of cells with OT and OTPP intensified the apoptosis process in a concentration-dependent manner compared to DATPP. The highest percentage of apoptotic cells was observed at the highest concentrations of OT and OTPP, while DATPP increased the apoptotic cells only by a much lower number. Simultaneously, after incubation with OT and OTPP, we observed a significantly reduced number of live cells (about 50%) in comparison to control and DATPP variants.

In conclusion, despite the stronger inhibitory properties of DATPP compared to OTPP, in addition to PDHC, oxythiamine and its diphosphate ester OT exhibited a much stronger cytostatic effect.

## Discussion

PDHC is one of the key enzymes of glucose metabolism^26^. The *K_m_* value of TPP in relation to PDHC determined in our study was similar to the value of the enzyme from bovine heart^27^ but ten times lower than the *K_m_* of the enzyme from European bison heart^28^. In the presence of OTPP, the *K_m_* value of TPP increased by about 50%. The relation between the *K_m_* value of TPP and the *K_i_* value of OTPP was similar to the data obtained in studies on bovine heart^27^ (*K_i_* of OTPP was about two times lower than *K_m_* of TPP). Referring to the *K_i_* values of OTPP obtained in our studies (0.025 µM) with the *K_i_* values reported in several other papers^12^^,^^27^^,^^28^ it was found that the value was lowest among those measured for PDHC from European bison heart^28^, bovine adrenals^12^ and bovine heart^27^ (Table 3). In the light of our results and the data obtained by other authors, we can state that PDHC from the porcine heart was the most sensitive to OTPP among the other studied sources. Experiments done on yeast-like fungi (*M. pachydermatis*, *C. albicans* and *S. cerevisiae*) showed that among the thiamine derivatives OT is the best inhibitor of cells growth and affects the activity of pyruvate decarboxylase and malate dehydrogenase^5^.

**Table 3.** Comparison of the literature *K*_m_ values of thiamine pyrophosphate (TPP) and *K_i_* values of oxythiamine pyrophosphate (OTPP) from different tissues (PDHC).

Source of tissue	*K*_m_ values of TPP (µM)	*K_i_* values of OTPP (µM)	References
European bison heart	0.6	0.23	Strumilo et al.^28^
Bovine adrenals	0.11	0.07	Strumiło et al.^12^
Bovine heart	0.07	0.04	Strumilo et al.^27^

Data obtained by Mann et al.^11^ showed that DATPP is the most potent among the known inhibitors of several TPP-dependent enzymes, including pyruvate decarboxylase complex from *Zymomonas mobilis* (*K_i_* = 14 pM) and α-ketoglutarate dehydrogenase from *E. coli* (*K_i_* = 5 nM). To our knowledge, no study has been performed analysing the impact of DATPP on PDHC isolated from mammals, but based on previously mentioned data, we can assume that a similar reaction may be observed on TPP-dependent enzymes from mammals. The results obtained by us for the PDHC from porcine heart (*K_i_* = 26 nM for OTPP) showed that the inhibitory potential of DATPP is lower than in case of microorganisms’ TPP-dependent enzymes, but it is still high. In contrast to the method used by Mann et al.^11^ which involved the inactivation of the enzyme in time under the influence of different DATPP concentrations, we used a kinetic method based on measuring the degree of PDHC inhibition in the presence of an increasing concentration of TPP at a constant concentration of DATPP (0.01 μM) added together with coenzyme and involving standard preincubation with the enzyme before starting the reaction of the substrate.

Our results demonstrate that in the case of PDHC, DATPP as well as OTPP is a competitive inhibitors, which is expected from other results concerning the effect of OTPP on other thiamine-dependent enzymes^5^^,^^11^. Comparing the *K_m_* values of TPP with the *K_i_* values of the tested antivitamins, we can define DATPP as a better inhibitor of PDHC than OTPP (*K_i_* values about 10 times lower). The better inhibition of PDHC by DAT may be related to the structure of this compound. The lack of nitrogen atom in the thiazolium ring may prevent ylide formation more effectively than the lack of an amino group in the case of OTPP. The theoretical chemistry data show that both the mentioned compounds can bind to the active centre of TPP-dependent enzymes with similar docking energy, which is lower in comparison with the native coenzyme^3^.

The data obtained for PDHC in the presence of OTPP (Table 3) showed higher values of *K_i_* in comparison with DATPP. The effect of DATPP on *in vitro* cell cultures has not been tested till now. Therefore, in our studies, we compared the effects of DATPP and OTPP as well as OT on HeLa cells. Based on the above mentioned enzymological data, we hypothesised that DATPP may reduce the rate of cell growth *in vitro* and limit the viability of HeLa cells, by more effectively inhibiting the TPP-dependent enzymes than OT or OTPP.

The *in vitro* test using HeLa cells showed that OT and OTPP had a stronger cytostatic effect in comparison with DATPP. Considering this, our initial hypothesis should be rejected.

Despite the strong inhibitory properties of DATPP in relation to PDHC, OTPP proved to be a stronger inhibitor of the growth of HeLa cells. The medium used in this study was carefully chosen to minimise the exogenous source of thiamine and to prove the impact of the chosen thiamine antivitamins independently. The results showed that the most effective as a cytotoxic compound was OT and its pyrophosphate. The reason for the lower sensitivity of cells to DAT may be the inability to transport the compound. Assuming, that thiamine and its derivatives are transported by the same proteins, lack of effectiveness of DATPP as a cytotoxic compound may result from several mechanisms.

SLC19A1 is a transporter responsible for the transfer of thiamine monophosphate across the cell membrane^26^^,^^29^^,^^30^. Research done by Mkrtchyan et al.^31^ showed that N2A cells had about five times higher amount of *SLC25A19* mRNA than astrocytes, but at the same time they exhibited similar expression of genes encoding *SLC19A2*^31^. However, in some other cancer cells, the expression of genes encoding transporters (such as *SLC19A3, SLC19A2*)^26^^,^^32–34^ is higher than in normal cells. Moreover, some cancer cells show higher expression of genes encoding *SLC25A19* transporter^26^, which may indicate a significant role of this transporter in the availability of thiamine phosphates. All the mentioned data suggest that the specific reaction of HeLa cells in the presence of thiamine derivatives may be related to the expression of genes encoding those transporters as well as the specificity of these transporters.

OT and OTPP are soluble in water, and so their dephosphorylation, transport and phosphorylation inside the cells may have no impact on their action in contrast to DATPP. Based on our knowledge, DAT is insoluble in water. Therefore, dephosphorylation of DATPP leads to release-free DAT outside the cell and thus could be the other reason responsible for the slight impact of this compound on HeLa cells.

The impact of OT on eukaryotic cells was studied on mammals (*in vitro* and *in vivo)* as well as on yeast. Analysis of the amount of cells of *S. cerevisiae* cells showed that OT reduced the total amount of cells^6^. Research done on mice showed the impact of the OT on the Ehrlich’s tumour^35^, and this observation was similar to the findings of our research. Data on the impact on fibroblasts showed no differences between the viability of cells with increasing concentrations of OT after 24 and 48 h^36^. Our research was maintained for approximately 4 days, what may play a role in the viability of cells. These suggest that fibroblasts are less sensitive than HeLa cells to OT treatment. Research done on MIA PaCa-2 cells in *in vitro* conditions showed that after exposure of cells to OT, their RNA content was reduced by about 45%, as well as the total amount of DNA (decreased by 20%)^37^. Moreover, in the same study, cell proliferation was inhibited by 31 and 41% at OT concentrations of 10^−8^ × 5 µM and 10^−7^ × 5 µM OT, respectively^37^. Moreover, proteomic studies on MIA PaCa-2 cells after exposure to OT showed that the amount of transketolase in cells was lower compared to the control group^38^. In addition, their data^38^ showed that the inhibition of transketolase by OT may have a wider impact on the cancer cells (such as activation of the apoptosis pathway). Our experiment done on HeLa cells confirmed the inhibitory properties of OT as well as OTPP.

From the results, we can summarise that DATPP has no significant impact on HeLa cells unlike OT. As we mentioned previously, the transport of DAT may be difficult. Its availability inside cells might be improved by preparing specific liposomes carrying DATPP or with the use of specific drug carriers^39^.

In light of our study, we can say that OT has a better cytostatic effect on HeLa cells than DAT. However, from the knowledge gained about the impact of DATPP on the kinetics of PDHC, we conclude that improving accessibility to the cells by the use of alternative transporters can improve the cytostatic effect of DATPP.
